# Recent advances in anti-multidrug resistance for nano-drug delivery system

**DOI:** 10.1080/10717544.2022.2079771

**Published:** 2022-05-26

**Authors:** Changduo Wang, Fashun Li, Tianao Zhang, Min Yu, Yong Sun

**Affiliations:** Department of Pharmaceutics, School of Pharmacy, Qingdao University, Qingdao, China

**Keywords:** Multidrug resistance, nano-drug delivery system, nanoparticles

## Abstract

Chemotherapy for tumors occasionally results in drug resistance, which is the major reason for the treatment failure. Higher drug doses could improve the therapeutic effect, but higher toxicity limits the further treatment. For overcoming drug resistance, functional nano-drug delivery system (NDDS) has been explored to sensitize the anticancer drugs and decrease its side effects, which are applied in combating multidrug resistance (MDR) via a variety of mechanisms including bypassing drug efflux, controlling drug release, and disturbing metabolism. This review starts with a brief report on the major MDR causes. Furthermore, we searched the papers from NDDS and introduced the recent advances in sensitizing the chemotherapeutic drugs against MDR tumors. Finally, we concluded that the NDDS was based on several mechanisms, and we looked forward to the future in this field.

## 1. Introduction

**Figure 1.**
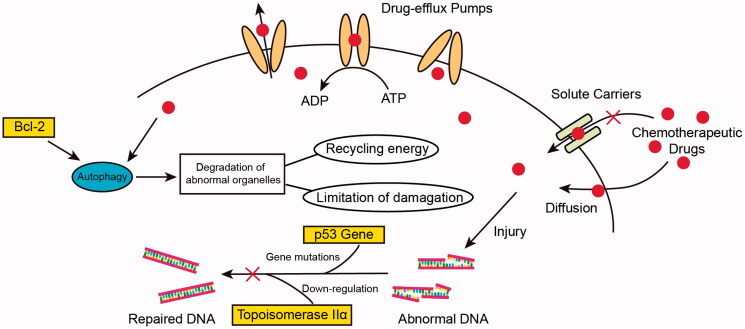
The main mechanism of tumor MDR. (i) overexpression of drug efflux pumps (ii) mutation of tumor suppressor genes such as p53 (iii) decreasing of influx by solute carriers (iv) modulated levels of enzymes like topoisomerase IIα (v) self-protected metabolism process such as autophagy.

Multidrug resistance (MDR) is the major cancer chemotherapy obstacle in clinics, which seriously disturbs the chemotherapy efficacy or even leads to failure. Higher chemotherapy drug doses were commonly applied to treat MDR-acquired cancer, but high toxicity and its adverse effects caused by higher drug doses impaired the healthy organs and tissues. The MDR development is complex and multifactorial. Drug-resistant tumor cells could decrease the intercellular drug concentration (or activity) via drug efflux pumps or intracellular microenvironment alteration. In addition, the intercellular signal pathway change would prevent tumor cells from apoptosis. The major MDR mechanism is overexpression of drug efflux pump, which could contribute to tumor resistance against various chemotherapeutic drugs including Adriamycin, Taxol, Carboplatin, Imatinib, and so on (Singh et al., 2017). It has been proven to be an effective strategy for MDR that increases intercellular drug concentration through circumventing ATP-binding cassette (ABC) transporters-mediated drug efflux (Figure 1).

There are many other mechanisms, except drug efflux pump, that would be closely associated with MDR. For instance, MDR-related genes participate in deoxyribonucleic acid (DNA) repairing, drug targeting alterations and drug uptake reduction through solute carriers. In previous studies, p53 plays an important role in drug resistance as a tumor suppressor gene. In addition, p53 mutations would engender the apoptosis disturbance and DNA repair mechanisms, which could resist DNA damage and apoptosis-induced damage caused by chemotherapeutic drugs (Ye et al., 2016). The DNA topoisomerase Iiα (topo IIα) involved in DNA replication and topo IIα downregulation is also regarded as a drug resistance mechanism (Wilson et al., 2001). Recent evidences have shown that autophagy is a double-edged sword for MDR tumors. Inducing autophagy, as a predeath process, could promote tumor cells apoptosis for overcoming MDR. What is more, inhibiting autophagy, as a presurvival process, could be resensitive to chemotherapeutic drugs in MDR cell (Li et al., 2017b).

## 2. Circumventing the drug-resistant cancer cell drug efflux

The ABC transporter family plays significant roles in MDR through decreasing intercellular drug concentrations as drug efflux pump, including P-glycoprotein (P-gp/ABCB1) (first identified ABC transporter), multidrug-resistant protein1 (MRP1/ABCC1), breast cancer–resistant protein (BCRP/ABCG2/MXR/ABCP), and multidrug-resistant protein 10 (ABCC10/MRP7) transporters (Li et al., 2017b). The P-gp, as a transmembrane efflux pump, discharges drugs from cytoplasm to the extracellular domain, causing the intercellular drug concentration reduction and the chemotherapeutic availability suppression. There are studies demonstrating that MDR could be potentially overcome by circumventing P-gp-mediated drug efflux with nano-drug delivery system (NDDS), such as nanotube, micelle, liposome, and nanometal material. The NDDS utilizes smart and special mechanisms to achieve drug release in designated circumstances (such as pH, hypoxia, and reducibility) or drug accumulation in tumors via passive targeting (EPR effect) or active targeting (ligand-receptor binding) (Li et al., 2021c; Zhou et al., 2020).

Meanwhile, nanoparticles could carry chemotherapeutic drugs into the cell through the endosome-lysosome pathway rather than passive diffusion, which has been wildly applied in reversing MDR (Wang et al., 2016b). Some substances (or groups) could bind to glycoprotein or lipoprotein on the cell membrane surface, inducing the plasma members’ curvature to the early endosomes formation that coat nanoparticles or other cargo and deliver them into cell. The hyaluronic acid (HA) and its derivatives can bind to the CD44 transmembrane protein family, which not only enhances drug internalization but also achieves targeted delivery. As a result of the superior biocompatibility, biodegradability, and non-immunogenicity of hyaluronan, HA is generally applied as a hydrophilic part of amphiphilicity micelles or is used as shell in coating nanoparticles. The tetrahydrofolic acid, derived from folic acid (FA), is involved in the one-carbon metabolism and plays an important role in the DNA replication. Thus, FA receptors overexpressed on the cancer cells members. The FA was modified on the nanoparticles surface and boosted NPs’ internalization against drug resistance (Beagan et al., 2020). Similarly, the cell-penetrating peptides could boost intracellular macromolecules delivery through receptor-independent endocytosis, which is used widely in the nanostructured systems. Low-molecular-weight protamine (LMWP), common CCPs, could overcome various biological barriers including the skin, mucosa, or tumor. Furthermore, it was used to modify nanoparticles for combating MDR through improving intratumoral delivery (Wang et al., 2014b). The enoxaparin sodium-modified poly(lactic-co-glycolic acid) (PLGA) hybrid carriers could enter into tumor cells by various endocytosis pathways, which showed a higher cytotoxicity in MCF-7/ADR cells as a result of circumventing the drug-resistant cancer cell drug efflux (Wang et al., 2016a). The tripeptide arginine-glycine-aspartic sequence (RGD) could bind preferentially to integrin αvβ3, and conjugation to iRGD significantly improved the tumor-imaging agents sensitivity and enhanced the antitumor drug activity.

**Figure 2.**
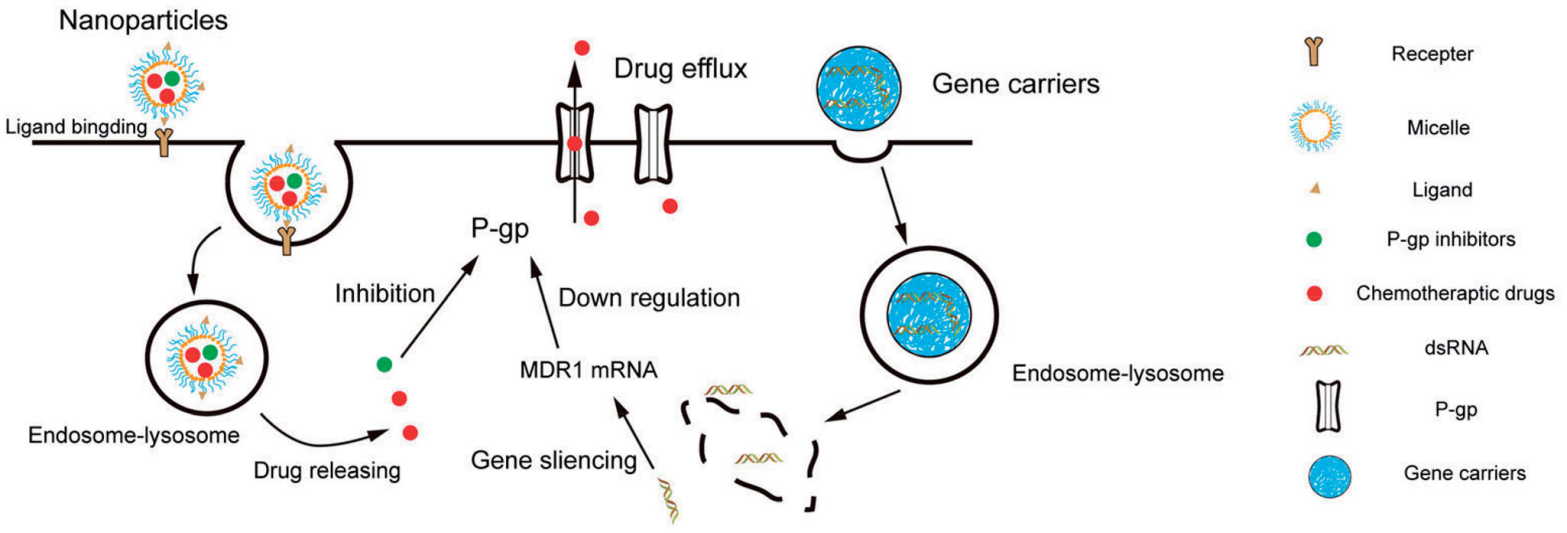
Overview of the strategies of circumventing the drug efflux in nano drug delivery systems. (i) enhance internalization mediated by NPs (ii) inhibiting P-gp by inhibitors (iii) silencing MDR1 mRNA via RNAi.

However, it is insufficient for MDR to simply enhance the drugs accumulation in tumor sites through the NDDS because of complex and multifactorial resistance, which inspires us to explore more insights into MDR (Figure 2 and Table 1).

**Table 1.** Examples of studies overcoming MDR reversing via bypassing drug efflux via endocytosis pathway.

Type	Chemotherapeutic drug	Mechanism	Cell line	Refs
PLGA hollow particles	DOX	Gas-generating agent targeted	MCF-7/ADR	(Ke et al., 2013)
PzLL-SS-PEG-SS-PzLLvehicles	DOX	Glutathione-mediated drug release	MDA-MB-231	(Ren et al., 2013)
LMWP-modified PLGA nanoparticles	DOX	Boost intracellular and intranuclear delivery	MCF-7/ADR	(Wang et al., 2014b)
Exosomes vehicles	PTX	Boost intracellular delivery	MDCK _MDR1_	(Kim et al., 2016)
enoxaparin sodium-PLGA nanocarriers	DOX	Boost intracellular and intranuclear delivery	MCF-7/ADR	(Wang et al., 2016a)
Pluronic-conjugated PAMAM dendrimers	DOX	Boost intranuclear delivery	MCF-7/ADR	(Wang et al., 2016b)
single-walled carbon nanotubes	EPI	CD44 receptor-mediated endocytosis	A549/ Taxol	(Yao et al., 2016)
HA-DOCA-His micelles	PTX	Endocytosis pathway	MCF-7/ADR	(Liu et al., 2018)
FA-poly(2-(diethylamino)ethyl methacrylate)	DOX	FA receptor-mediated endocytosis	MCF-7/ADR	(Beagan et al., 2020)
Mesoporous silica nanospheres	DOX	Via nonspecific endocytosis	MCF7/ADR	(Xu et al., 2018b)
PEG-PGC-PDLA	DOX	Boost intracellular delivery	Bats-72, Bads-200	(Zhong et al., 2019)
gold-silver nanorod&AuNPs	DOX	Nuclear-uptake mediated by aptamer	K562/D	(Qiu et al., 2015)
rTL/ABZ@BSA/Ag NP	Trichosanthin, Albendazole	Boost intranuclear delivery	A549/T, HCT8/ADR	(Tang et al., 2017)
Ir-Cb ADDC	Irinotecan, chlorambucil	Boost intracellular delivery	MCF-7/ADR	(Huang et al., 2014)
Lipid Nanoparticles	Edelfosine	Boost intracellular delivery	HL-60 & K-562	(Aznar et al., 2014)
cNC@PDA-PEG	PTX &lapatinib	Endocytosis pathway	MCF-7/ADR	(Wang et al., 2020b)

### 2.1. Small-molecule agents of P-gp inhibitors

**Figure 3.**
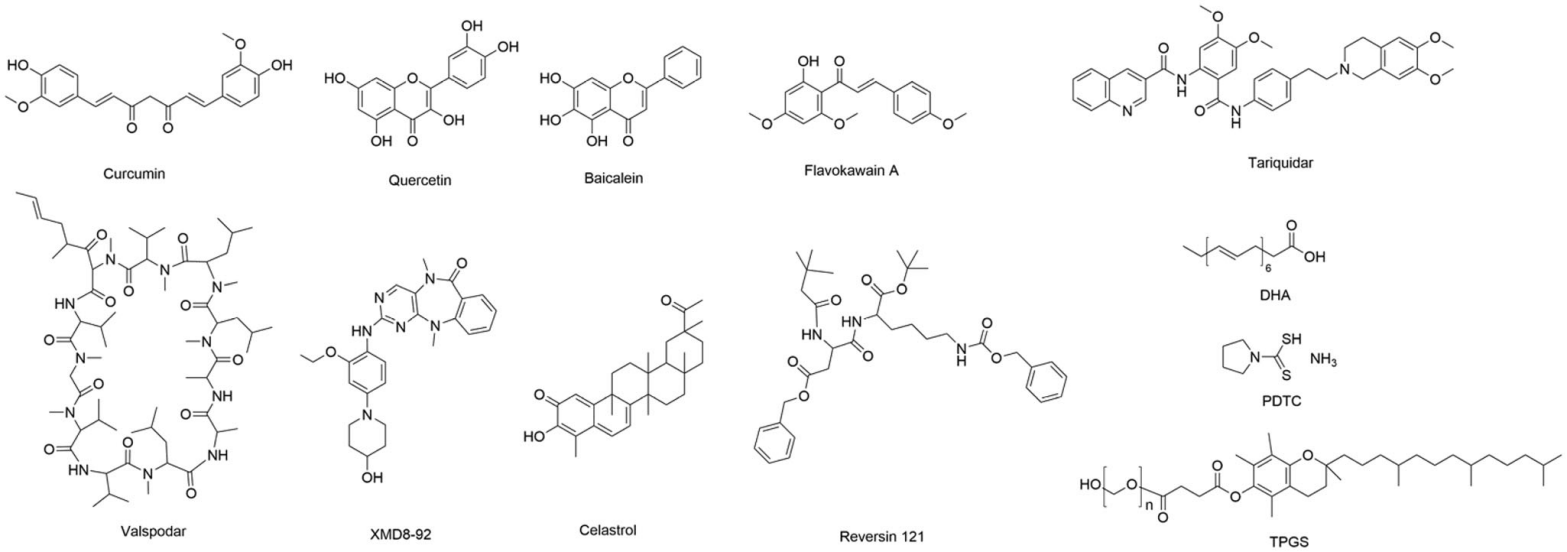
The chemical structural formula of small-molecule P-gp inhibitors in NDDS.

There are various small-molecule agents of P-gp inhibitors, such as curcumin and valspodar, which could directly regulate the P-gp expression or activity. However, they often cause chronic toxicity and systemic side effects. Thus, NDDS could take a multifunctional nanoparticles advantage to target the codelivery of different drugs toward the tumor site and could achieve a long-time blood circulation, drug accumulation in the tumor site, decreasing side effects, and enhancing the chemotherapy validity (Yee Kuen & Masarudin, 2022). Curcumin is an extract of traditional Chinese medicine *Curcuma longa L*. and can reverse the tumor MDR via regulating the MDR-related proteins (such as P-gp) and the MDR-related signal pathways (such as NF-κB) (Xu et al., 2020). The PLGA nanoparticles which coloaded curcumin and DOX can avoid the rapid drug leakage and had a stronger antitumor effect in DOX-resistant esophageal cancer cells (TE10/DOX) (Gao et al., 2021b). Quercetin can inhibit the P-gp expression and mutant p53 gene. Quercetin and DOX were encapsulated in mSiO_2_ nanoparticles coated with pH-sensitive polydopamine (PDA), which effectively showed reverse MDR of HCT-8/TAX cells (Shao et al., 2019). The nanosystems could be developed into various formations and more applications through modifying copolymers with functional groups or loading more different agents. Tariquidar is a specific ABC transporter inhibitor of P-gp. Zhen et al. encapsulated fluorophore and Tariquidar within polymeric prodrug for combined image-guided PDT and drug-resistant cancer chemotherapy (Zhen et al., 2019). In addition, Tariquidar can also be embedded into CSCs-specific targeted nanocarriers with DOX to overcome breast CSCs MDR (Pan et al., 2020). Through codelivering the pyrrolidinedithiocarbamate (PDTC) and DOX by pH-sensitive polymeric nanoparticle, it can inhibit the NF-κB nuclear translocation and downregulate the P-gp expression, thus improving the intracellular drugs accumulation (and penetration) and enhancing DOX-induced cytotoxicity and apoptosis in MCF-7/ADR cells (Cheng et al., 2019). It is also a smart strategy that cytostatic drug and P-gp inhibitor covalently bound together to the same polymeric carrier through a degradable bond, which shows excellent potency in overcoming natural MDR (Sivak et al., 2017) (Figure 3).

The P-gp modulator valspodar is combined with ultrasound-induced hyperthermia (USHT) to enhance the cellular retention and cytotoxicity of rhodamine 123 and DOX, which could effectively reverse MDR (Liu et al., 2001). The XMD8-92, as a big mitogen-activated protein kinase 1 (BMK1) inhibitor to target p53 upregulation, could efficiently downregulate the P-gp expression. Yang et al. designed targeting NDDS to codeliver the DOX, XMD8-92, and SPIOs, which combined chemotherapy with magnetic resonance (MR) imaging and exhibited an outstanding cytotoxicity on MDR cells (Yang et al., 2019). Disulfiram, an apoptosis inducer and P-gp inhibitor, was codelivered with DOX and used to reverse MDR (Duan et al., 2013).

Several nonionic surfactants have showed the functions of inhibiting the P-gp efflux pump and could regulate the pharmacokinetics and cellular transport of drug, which has been applied in combating the tumor MDR. The TPGS (D-α-tocopherol acid polyethylene glycol succinate) is a water-soluble vitamin E derivative. Recent evidences suggest that the TPGS could reverse the MDR by inhibiting the ATPase activity without the influence of intracellular adenosine 5′-triphophagte (ATP) or mitochondrial function (Hou et al., 2019). Considering that the TPGS is an excellent nonionic surfactant, it could be used to stabilize and modify several nanoparticles. Mesoporous silica nanoparticles (MSNs) have been widely applied in the NDDS because of their large specific surface area, excellent biocompatibility, and good dispersibility in water. The MSNs surface was modified by the TPGS for loading DOX to overcome MDR, which revealed noticeable antitumor efficacy in drug-resistant breast tumor (Zhao et al., 2018). Similarly, the TPGS also was used as a surface modifier to functionalize upconversion nanoparticles which unfolded several excellent properties including water solubility, P-gp inhibition, and image guidance. Furthermore, this system was able to overcome MDR obviously in MCF-7/DOX (Tian et al., 2015). Except in simple surface modification, the TPGS could also covalently graft on the surface of poly(amidoamine) dendrimers to loading DOX and carbon dot, which improve the ultrasound-enhanced fluorescence imaging and chemotherapy to overcome MDR (Li et al., 2021a). Hou et al. conjugated HA with vitamin E succinate as self-assemble micelles for reversing MDR, which not only achieve CD44 receptor overexpressing tumor targeting but also retain the inhibition of P-gp (Hou et al., 2019). Docosahexaenoic acid (DHA) is one of the w-3 unsaturated fatty acid and can chemosensitize MDR. The DHA, as functional excipient, was used to synthesize nanoparticles for reversing drug resistance in hepatocellular carcinoma (HCC) via suppressing drug-resistant proteins MRP, LRP, BCRP, and Bcl-2 (Wang et al., 2021a).

### 2.2. Small interfering ribonucleic acid

The sequence-specific gene silencing offers a relatively safe method for P-gp downregulation without side effects compared with small-molecule agents of P-gp inhibitors. Obviously, it is an efficacious strategy for MDR to targeting P-gp coded by the MDR1 gene (Tariq et al., 2020). Ribonucleic acid (RNA) interference (RNAi) takes an advantage of small interfering RNA (siRNA) molecules to silence specific genes and regular gene expression, which has presented an immense potential in anticancer research as well as overcoming MDR. Double-stranded RNA (dsRNA) firstly cleaves by the enzyme DICER into smaller special length and structure segments, known as siRNA, which will be embedded into the RNA-induced silencing complex (RISC), and then unwinds the double-stranded siRNA into single-stranded segments. With the SiRNA guide, the RISC complex could cleave and degrade targeted mRNA under the Ago2 protein enzymolysis to achieve the targeted gene silencing (Horak, 2020).

Nucleic acids are anionic biomacromolecules; thus, cationic lipid and polymer systems can electrostatically bind to RNA and facilitate the gene transfection. In previous reports, a number of functional vehicles have been widely used to achieve the nucleic acid and anticancer drugs simultaneous delivery. Cationic polymers can be designed as hydrophilic segments and covalently linked to hydrophobic groups, which do not only achieve chemotherapeutic drugs and siRNA codelivery but also further modify amphiphilic copolymer to possess more functions (Pan et al., 2019; Shen et al., 2014). Molecular beacons, which are widely used for molecular targets detection and imaging in living cells, were loaded in graphene oxide carriers with DOX and hybridized with the target mRNAs for silencing MDR1 mRNA and upstream ETS1 mRNA, which shows effective a P-gp expression inhibition and further reverse MDR (Li et al., 2018).

## 3. Disrupting energy metabolism or signal pathways

Considering that the drug efflux pumps, such as P-gp, require energy from ATP hydrolysis, one of the strategies for MDR is the energy supply interdiction to P-gp. There are several ways in inhibiting P-gp from the energy supply, which include the inhibiting energy metabolism, decreasing energy production, and blocking signal pathways mediated by ions.

### 3.1. Targeting mitochondria

**Figure 4.**
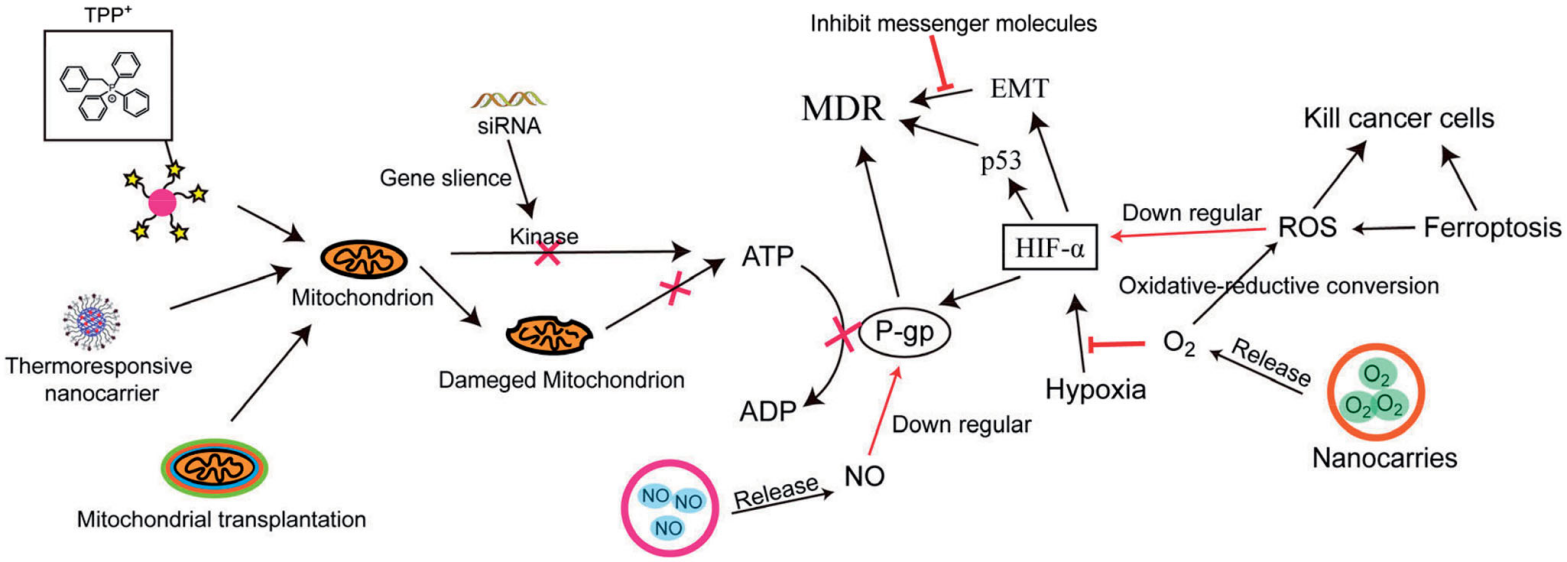
General overview of targeting cell energy metabolism to overcome MDR. (i) disturbing the function of the mitochondrion disturbing by NPs or mitochondrial transplantation (ii) Disturbing the intracellular microenvironment (iii) NO suppressing P-gp activity.

Mitochondria are the organelle producing energy in cells; thus, disturbing its function could be used in overcoming MDR. Compared with normal or non-MDR cells, MDR cells have higher polarized mitochondrial membranes which are related to drug efflux dependent on ATP. Triphenyl phosphonium cation (TPP) is a mitochondria targeting group which is frequently applied in the delocalized lipophilic cations. Wang et al. modified the TPP into PEO-PPO-PEO triblock copolymer which not only easily internalized into tumor cells but also reversed MDR through facilitating TPP mitochondria targeting (Wang et al., 2020a). The biomedical strategy based on mitochondria is a novel means to overcome MDR and supplies a way to fight nonpump factors in MDR (Chen et al., 2021a). Currently, mitochondrial transplantation has been applied to enhance drug sensitivity and combat MDR, which can affect organelle functions through disordering energy metabolism. Chen et al. developed a nanomaterial-coated mitochondria complex to overcome MDR caused by mitochondrion-mediated nonpump factors, while also modifying the mitochondrial surface with polycations (chitosan and poly[acrylic acid]) and layer-by-layer technique to load MDR-siRNA (Chen et al., 2019b). Pyruvate kinase M2 (PKM2) is a glycolytic pyruvate kinase isoenzyme that produces ATP, which is overexpressed in malignant tumor cells and supplied energy to ABC transports. The PKM2 siRNA and DOX were loaded in PAMAM and were selectively delivered to tumor. In addition, the PKM2 inhibition could facilitate intracellular DOX effects against A549/ADR cell (Shu et al., 2021). The active mitochondria can maintain higher temperature (approximately 48 °C), which supplied the strategy of mitochondria to be temperature-responsive for reversing drug resistance in lung cancer. Thermoresponsive nanocarrier poly (N-isopropylacrylamide) (PNIPAM) was used for mitochondria-targeted delivery against DOX-resistant small-cell lung cancer (H69AR) (Ruan et al., 2022) (Figure 4 and Tables 2 and 3).

**Table 2.** Examples of studies overcoming MDR reversing via co-delivering chemotherapeutic drug and P-gp inhibitors.

Type	Chemotherapeutic drug	Mechanism	Cell line	Refs
PLGA nanoparticles	DOX	Curcumin	TE10/DOX	(Gao et al., 2021b)
HMSNs-PDA-PEG	DOX	Quercetin	HCT-8/TAX	(Shao et al., 2019)
mSiO_2_ -dPG	DOX	Tariquidar	MDA-MB-231	(Pan et al., 2020)
poly(ortho ester urethanes) copolymers	DOX	Pyrrolidinedithiocarbamate	MCF-7/ADR	(Cheng et al., 2019)
HPMA copolymer conjugates	DOX	Reversin 121	P388/MDR	(Sivak et al., 2017)
PEG-b-Leu micelles	DOX	XMD8-92 (BMK1inhibitor)	SCG 7901/VCR	(Yang et al., 2019)
HA conjugated VES polymer	PTX	VE succinate	MCF-7/ADR	(Hou et al., 2019)
mesoporous silica nanoparticles	DOX	TPGS group	MCF7/MDR	(Zhao et al., 2018)
Up-conversion nanoparticles	DOX	TPGS group	MCF7/ADR	(Tian et al., 2015)
G5-TPGS@y-CDs	DOX	TPGS	MCF-7/ADR	(Li et al., 2021a)
DHA-nanoparticles	DOX	DHA	HepG2/DOX	(Wang et al., 2021a)
Cetuximab chitosan nanoparticles	PTX	Quercetin	A549/Taxol	(Chen et al., 2021a)
Nanoemulsions	PTX	Baicalein	MCF-7/Tax	(Meng et al., 2016)
PLGA-based NPs	PTX	Blocking calcium channels by verapamil	MCF-7/ADR	(Afrooz et al., 2017)
LyP-1 LMWH-Qu conjugate	Gambogic acid	Quercetin	MCF-7 (overexpressing p32)	(Tian et al., 2018)
drug-drug conjugate	DOX	Celastrol	MCF-7/ADR	(Xiao et al., 2018)
VES-GFLG tetra peptide-Chitosan	PTX-VE	TPGS group	MDA-MB-231/PTX	(Zhang et al., 2018a)
PCL_200_ /PCL_5_nanoparticles	DTX or PTX	MePEG _17_ -b-PCL_5_ (P-gp inhibitor)	MDCK-MDR	(Jackson et al., 2020)
MSNs-ChS@PQ	PTX	Quercetin	MCF-7/ADR	(Liu et al., 2020b)
DOX-AuNR-PCDA-PEG-Biotin	DOX	Curcumin	MCF-7/ADR	(Wang et al., 2018)
Aes-stabilized nanoparticles	PTX	flavokawain A	A549/T	(Li et al., 2020)
Cur-pCB-Dox	DOX	Curcumin	MCF-7/ADR	(Zhao et al., 2020)

**Table 3.** Examples of studies silencing P-gp related gene by siRNA.

Type	Chemotherapeutic drug	Mechanism	Cell line	Refs
PEI-CyD-cholesterol Micelles	DOX	MDR1 siRNA and shRNA	MCF-7/ADR	(Shen et al., 2014)
poly (b-amino esters)	DOX	Mdr-1-shRNA and Survivin-shRNA	MCF7/ADR	(Yin et al., 2012)
polyamidoamine-modified selenium nanoparticles	Cisplatin	MDR1 siRNA	A549/DDP	(Zheng et al., 2015)
thiolated glycol chitosan polymers	DOX	self-polymerized Mdr1-siRNA	MCF-7/ADR	(Yhee et al., 2015)
molecular beacon (MB)-based micelles	DOX	MDR1 MB	OVCAR8/ADR	(Zhang et al., 2017)
graphene oxide (GO)	DOX	MDR1 MB and ETS1 MB	MCF-7/ADR	(Li et al., 2018)
CS-PEI	PTX	Beclin-siRNA	NCI-H23-TXR	(Liu et al., 2019b)
G4-PAMAM-PEG-DOPE	DOX	MDR1 siRNA	MCF7/ADR	(Pan et al., 2019)
erythrocyte-derivedmimic vesicles	DOX	MDR1 siRNA	MCF-7/R	(Wang et al., 2019d)
PLNP-PDA-PEI-FA-DOX-siRNA	DOX	MDR1 siRNA	MCF-7/ADR	(Su et al., 2019)

### 3.2. Disturbing the intracellular microenvironment

Due to the rapid growth of cancer cells, most of the solid tumors could cause specific microenvironment that differs from the normal tissues, such as hypoxia. Tumor cells are able to constantly adapt to this specific microenvironment and cause a metabolism imbalance, which further affects the chemotherapeutic drugs therapy. Hypoxia-inducible factor-1α (HIF-1α), upregulated at a low-oxygen concentration, is closely related with drug resistance through modulating P-gp or p53 gene. Although it is a way to combat hypoxia-induced resistance to oxygen delivered to tumor tissues, the oxygen supplement also offers more vitality to tumor cells. Thus, the solutions to hypoxia-induced resistance are the general need for synergetic therapy (Zhuang et al., 2021). Hypoxia counts against body cell mitosis as well as tumor cells, but hypoxia could promote epithelial-mesenchymal transition (EMT) which can accelerate tumor metastasis. Ma et al. utilized high-intensity focused ultrasound to trigger releasing DOX and oxygen inside the tumor to alleviate MDR, leading to excellent tumor inhibition activity compared with free drug therapy (Ma et al., 2020). The hepatic stellate cells (HSCs) activation can also induce EMT by secreting various messenger molecules, such as substance P (SP), and then promote the drug resistance of hepatocellular carcinoma (HCC). The therapy strategy based on capsaicin (CAP, an inhibition of SP) and DOX can inhibit the HSCs activation, inhibit the cancer metastasis, and suppress the HCC drug resistance (Li et al., 2021e). Moreover, further decreasing oxygen (O_2_) in cancer cells can create starvation effects for resensitizing chemotherapeutic drugs in MDR tumors. Zhang et al. designed a nanoconjugate based on AuNPs to convert glucose and O_2_ into gluconic acid and hydrogen peroxide (H_2_O_2_), which causes harsh environment of hypoxia-associated acidic acid and induces the cancer cells apoptosis (Zhang et al., 2020). Wei et al. synthesized a polymer with diselenium bond for encapsulating Pt (IV) prodrugs (NP[Se]s), which can effectively break the intracellular redox balance via GSH depletion and reactive oxygen species (ROS) generation for platinum-based cancer therapy (Wei et al., 2020).

### 3.3. Inducing ferroptosis

Ferroptosis is an iron-dependent programmed cell death through catalyzing the excessive hydroperoxides of membrane polyunsaturated fatty acid to induce cell death. Fu et al. integrated hollow mesoporous silica (HMS)-based nanoplatform with oxidative ferrate (Fe[VI]) and then incorporated n-heneicosane (HE) and PEG (named as DOX-Fe[VI]@HMS-HE-PEG). This nanoplatform can lead to oxygen species overproduction and suppress the glutathione peroxidase 4 (GPX 4) (a critical regulatory target of ferroptosis), which shows excellent inhibition to drug-resistant hypoxic tumors (Fu et al., 2021). Besides, the highly toxic hydroxyl radical (OH) can injure the intracellular biomolecules and kill drug-resistant tumors, which are known as chemodynamic therapy. The DOX-loaded Fe-porphyrin COF nanoparticles (DOX@COF[Fe]) were designed to convert intracellular hydrogen peroxide (H_2_O_2_) into ·OH via Fenton-like reaction against adriacin-resistance MCF-7 cells (Gao et al., 2021a). Gallic acid-ferrous (GA-Fe(II)) has strong catalytic ability to catalyze H_2_O_2_ into ·OH. In addition, overgenerated ·OH can promote lipid peroxidation (LPO) and ferroptosis to achieve tumor-killing efficacy in MCF-7/ADR cells (Zheng et al., 2022).

### 3.4. Distributing signal pathways

Nitric oxide (NO), as a biological signal molecule, plays an important role in several vital movements. High NO concentrations induce tumor cell apoptosis and death as well as suppress P-gp expression for reversing the cancer cells MDR (Wang et al., 2019a). Nanosystems focusing on releasing NO have been developed for reversing MDR in combination with chemotherapy. The NO gas was hard to be directly delivered to tumor tissue due to extremely short half-life and instability; thus, different kinds of NO donors (such as N-diazeniumdiolates [NOates] and S-nitrosothiols [RSNO]), which were integrated with polymers or other inorganic materials, were needed to achieve the triggered NO release. The RSNO were conjugated with poly(thlene glycol)-block-poly(propylene sulfide) amphiphilic copolymer for encapsulating DOX, which was unable to respectively release DOX and NO by oxidative-reductive conversion (Wu et al., 2020). Similarly, NO donor and DOX were conjugated to poly(amidoamine) (PAMAM) via acid-cleavable cross-link and then were further masked by modified PEG, which could release NO to reverse hypoxia-induced drug resistance (Wang et al., 2022). The NO-responsive liposome was designed to NIR-trigger drug release and inhibits P-gp in combination with Au-nanorod-embedded CuS shell, allowing NO and DOX release in drug-resistant tumor cells to kill MDR cancer cells (Wang et al., 2019a). The TPGS-3NO_3_, as a nitric donor, was involved in nanomicelles and targeted to deliver to tumor tissue via sialic acid receptor, which can reverse cisplatin (CDDP) resistance as well as enhance antimetastatic efficacy in hypoxia cancer cells (Chen et al., 2021b).

In addition, among cyclic lactate catabolism, lactate dehydrogenase A (LDHA) can promote the tumor cells chemotherapy resistance. An intelligent bioreactor was tailor-made by the integration of metal-organic framework (MOF) with nonpathogenic *Shewanella oneidensis* MR-1 (SO), and the lactic acid catabolism consumed by SO and MOF could suppress the P-gp expression which could effectively conquer drug-resistant tumors (Wang et al., 2021c). Considering the let-7 family negatively regulating proto-oncogenes, cyanine5 (Cy5)-modified miRNA (let-7i) and platinum were integrated into nanographene oxide for reversing drug resistance via suppressing the cyclin D1 protein (Yan et al., 2022). The miRlet-7a and PTX were coloaded within MSNs-functionalized gold nanorods to overcome ovarian cancer treatment MDR (Wang et al., 2021d).

### 3.5. Distributing ion homeostasis

Ion homeostasis in cells is closely related with intracellular energy metabolism and further affects the tumor drug efflux pump activity. Adjudin, a chloride ion channel blocker, could affect mitochondria function that is initially used for researching sperm activity and contraception. Furthermore, recent studies showed that it could restrain tumor growth and be applied in reversing MDR (Wang et al., 2019c). Tumor cells are more sensitive to Ca^2+^ compared with normal cells because it lacks a sufficient Ca^2+^ metabolism pathway; meanwhile, intracellular Ca^2+^ overload can disturb the tricarboxylic acid cycle, reduce the oxygen consumption, and suppress the overexpression of P-gp caused by downregulating HIF-1α. Liu et al. prepared a tumor targeting “calcium ion nanogenerator” to deliver Ca^2+^ and DOX into tumor cells and reverse MDR through an intracellular Ca^2+^ bursting strategy (Liu et al., 2020a). Due to excessive Ca^2+^ providing a survival pathway for cancer cells, higher cellular Ca^2+^ is generally recognized as an important resistance characteristic. The phytic acid (PA)-modified CeO_2_ nanoinhibitors were designed to reverse MDR through an efficient inhibiting Ca^2+^ (Tian et al., 2022) (Table 4).

**Table 4.** Examples of studies overcoming MDR reversing via targeting energy metabolism.

Type	Chemotherapeutic drug	Mechanism	Cell line	Refs
Nanomaterial-coated mitochondria complex	DOX（alone iv）	Mitochondrial transplantation, MDR1siRNA	MCF-7/ADR	(Chen et al., 2019b)
DOX@PNIPAM	DOX	Mitochondrial temperature-responsive	H69AR	(Ruan et al., 2022)
TPP-PF127-HA/PTX micelles	PTX	TPP	A549/ADR	(Wang et al., 2020a)
TPP^+^-conjugated Brij 98	PTX	Triphenylphosphonium cation	MCF-7/ADR	(Han et al., 2019)
Spherical helical polypepetide	DOX	PKM2 siRNA	A549/ADR	(Shu et al., 2021)
Cerasomal perfluorocarbon nanodroplets	DOX	O_2_ (HIFU)	MDA-MB-231 (hypoxia)	(Ma et al., 2020)
CAP/GA-sHA-DOX NPs	DOX	Blocking “SP-HSCs-HCC” axis	BEL-7402, LX-2	(Li et al., 2021e)
AuNP-PEG-RGD-GOx	DOX	O_2_ (H_2_O_2_)	MCF-7R	(Zhang et al., 2020)
DOX-Fe(VI)@HMS-HE-PEG	DOX	Ferroptosis	Saos-2 (hypoxia)	(Fu et al., 2021)
SO@MIL-101-DOX-HA	DOX	Fe^3+^, acultative anaerobes	DOX-resistant 4T1	(Wang et al., 2021c)
DOX@COF[Fe]	DOX	·OH	MCF-7/ADR	(Gao et al., 2021a)
GA-Fe(II)/DOX@liposome	DOX	·OH	MCF-7/ADR	(Zheng et al., 2022)
pH-instable PDN Cluster	DOX	NO	4T1 (hypoxia)	(Wang et al., 2022)
PGE-PPS-GSNO nanoparticles	DOX	NO	HepG2/ADR	(Wu et al., 2020)
PEGylated nano-graohene oxide	Platinum (IV)	miRNA (let-7i)	SKOV_3_DDP	(Yan et al., 2022)
HA-PTX/let-7a-GNR@MSN	PTX	miRNA (let-7i)	SKOV3_TR_	(Wang et al., 2021d)
CePA	DOX	Phytic acid	HepG2/ADR	(Tian et al., 2022)
Calcium ion nanogenerator	DOX	Inducing intracellular Ca^2+^ bursting	MCF-7/ADR	(Liu et al., 2020a)

## 4. Phototherapy

**Figure 5.**
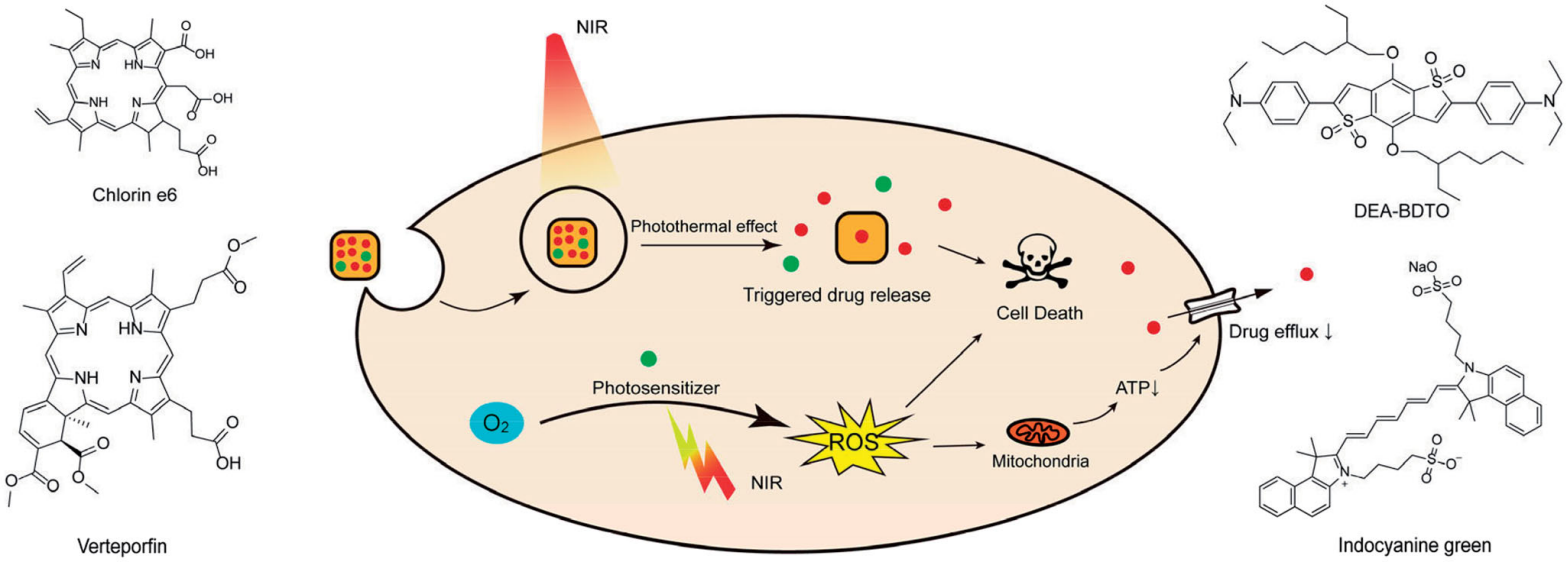
Overview of phototherapy to overcoming MDR in nano drug delivery systems.

Photothermal therapy (PTT), as well as photodynamic therapy (PDT), is a noninvasive treatment strategy based on special materials in response to special wavelength light, which has been widely applied in the cancer diagnosis and treatment (Li et al., 2017a; Zhang et al., 2021). Recently, it could enhance antitumor efficiency when chemotherapy is combined with PTT or PDT through nanotechnology, which not only achieves the antitumor drugs targeting releasing but also integrates the PTT or PDT tumor kills. So, more and more researches have focused on utilizing PTT or PDT to combat MDR (Li et al., 2021d). Near infrared (NIR) light has been the main illuminant for PTT and PDT because of the NIR advantages including deep tissue penetration and low tissue damage. PTT isn’t only directly kill cancer cells through thermal ablation, but also indirectly improve the effects of chemotherapy by controlling drug release (Huang et al., 2021). Gold nanoparticles (AuNPs) are hyperthermia agents that can realize photothermal conversion under NIR light. The AuNPs could be encapsulated within a DOX-loading multiporous shell, and DOX was accelerated to be released under the local high temperature via the 808-nm NIR light trigger. This drug delivery system could increase the drug dose but not cause the additional adverse effects; thus, it shows great capability to combating MDR in breast cancer (Wu et al., 2019). Similarly, copper selenide (Cu_2-x_Se), as a photothermal agent, has high photothermal conversion efficiency and is combined with phase change materials to achieve the rapid drug release with photothermal control. The Cu_2-x_Se nanoparticles encapsulating chemotherapeutic drugs could enhance drug release and retention to overcome MDR breast cancer and bypassing P-gp-mediated drug efflux compared with nonphotothermal vector (Ji et al., 2021). Fucoidan-decorated silica-carbon nano-onion hybrid nanoparticles target tumor vasculature to specifically release P-gp inhibitor and DOX at a low NIR power (Wang et al., 2021b). It seems to be hard to completely overcome MDR that just using PTT improves the intracellular concentrations of chemotherapeutic drugs (Figure 5).

PDT is based on photosensitizer to convert oxygen into cytotoxic-ROS under light irradiation (Hou et al., 2022). The mechanisms of photosensitizer generating ROS can be divided two types: (1) Photosensitizer transfers light energy to either tissue substrate and produce superoxide anion radicals (such as O_2_^−^ and HO_2_), which is called as Type I reaction; (2) Photosensitizer directly converts O_2_ molecules into singlet oxygen (^1^O_2_), which is Type II reaction (Aniogo et al., 2019). The ROS productions don’t only destroys tumor tissues but also decreases the HIF-1α expression (Pramual et al., 2020; Zhuang et al., 2021). Excessive ROS generated by photosensitizer could induce biomembrane lipid peroxidation and kill of cancer cells. Besides, ROS during PDT significantly disrupted mitochondria and decreased intracellular ATP, which would suppress drug-efflux to overcome MDR (Han et al., 2016). Considering several PDT advantages in anticancer treatment such as high targeting and minimal side effects, it was applied in combating MDR in breast cancer, lung cancer, gastric cancer, and oral malignancies.

Faceted Au NRs can generate abundant ROS due to high index facets, which can efficiently convert *L*-Arg into NO upon NIR laser activation. *L*-Arg/DOX-loaded gold@copper sulfide yolk-shell nanoparticles (ADAu@CuS YSNPs) could inhibit P-gp expression and enhance DOX accumulation in MCF-7/DOX (Wang et al., 2019a). Except gold nanoparticles, the Ru (II) complex also is a photosensitizer that can generate ^1^O_2_ under light irradiation, which exhibits excellent activities for MDR cancer (Cabrera-González et al., 2019). The bimetallic combination of Ru and Pt draw attention to the treatment of drug-resistant tumor, where Ru and Pt complement each other (Karges et al., 2020). Zeng et al. designed a Pt (IV)/Ru (II) bimetallic polymer which can generate ^1^O_2_ within 671 nm light and trigger Ru (II) anticancer agent after polymer degradation. At the same time, anticancer drug cisplatin is released from Pt (IV) moieties with GSH reduction response (Zeng et al., 2020). Porohyrinoid-based photosensitizers are the most common and abundance photosensitizers, which are the framework of chlorophyll (Pucci et al., 2021). The four nitrogen atoms of porphyrinioids can complex various metal cations, which will dramatically affect ROS generation (Otvagin et al., 2022). Targeted by mitochondria chimeric peptide, in situ PDT caused by protoporphyrin IX would reduce ATP and enhance DOX efficacy against drug-resistant tumor (Han et al., 2016). Chlorin e6 (Ce6) is a porohyrinoid-based photosensitizer which has been widely used in PDT. Glucose oxidase (GO_x_) and Ce6 were incorporated into PLGA/MOF nanoassembly, which can consume glucose and produce H_2_O_2_ to remodel multiple-resistant TME (Liu et al., 2021a). Ce6 was co-loaded in protein-based nanocarrier with docetaxel, which had a strong synergistic antitumor effect in DTX-resistant Hela cells (Gaio et al., 2019). In order to heighten the antitumor effect in PDT-resistant cells, Ce6 could selectively released from NPs via 4,4′-azodianiline bond (–N = N–, a hypoxia reactive linker) under hypoxia condition (Lee et al., 2022). Generally, light with longer wavelength has stronger tissue penetration. Upconversion nanoparticles can convert long wavelength light (such as 808 or 980 nm) into shorter wavelength light (such as 365, 475, 545, 655, or 800 nm), which is used to trigger the Ce6-containing nanocomposite to generate ^1^O_2_ for overcoming tumor MDR (Xu et al., 2018a). Verteporfin, a porphyrin photosensitizer, was conjugated with tumor angiogenesis-targeting iNGR peptide and TPGS to treat drug-resistant tumor via efficient photo-chemotherapy (Jiang et al., 2019). Some NIR fluorophores based on BDTO core also have a great ability to generate ROS; thus, Zhen et al. designed a polymeric PTX prodrug to load NIR fluorophores, as photosensitizer, and P-gp inhibition tariquidar against MDR cancer (Zhen et al., 2019). This study showed that the image-guided PDT and chemotherapy combination has better therapeutic effects than single-drug treatment. All in one polymeric agent (P1-EPO) was based on endoperoxide 1,4-dimethylanpthalene derivative (DMN-EPO) and halogenated aza-BODIPY, which can release ^1^O_2_ under 808 nm laser (Li et al., 2021b). The therapy based on photoabsorber-antibody conjugates, known as photoimmunotherapy, has been demonstrated that it could effectively suppress sicplatin-resistant SBC-3 cells (Takahashi et al., 2021).

What’s more, PDT could be integrated with PTT to overcome MDR together, which showed excellent effects. Indocyanine green (ICG) is a NIR dye and photosensitizer and has an advantage of integrating PTT with PDT, which could convert the NIR light into ROS and heat energy. The ICG is widely applied in enhancing antitumor therapy efficiency, demonstrating to be accumulated in the MCF-7/ADR cells nuclei (Wang et al., 2019b). The ICG and DOX coencapsulation within polymer nanoparticles could integrate chemotherapy and photothermal therapy, which improves the therapeutic efficacy against MDR tumors (Chen et al., 2019a). For enhancing the ICG and DOX antitumor efficacy on MDR breast cancer cells, ICG and DOX were embedded in PCM matrix of the AuNCs which can release drugs at high temperature by NIR light raying (Yu et al., 2017). Recently, Ding et al. developed multifunctional nanoparticles, composed with SNO-modified polymer micelles and poly(dopamine) shell, which integrates PTT, NO gas therapy, and chemotherapy for enhancing chemotherapy validity and combating MDR by PTT and NO (Ding et al., 2019). An X-ray, except infrared light, can activate photosensitizer to generate ROS as well. Selenadiazole derivatives (SEDs) can generate ROS under X-ray to induce DNA breakage and boost apoptosis, which were loaded in MSNs to enhance chemotherapy by affecting p52, protein kinase B, and mitogen-activated protein kinase pathway (Liu et al., 2021b). Similarly, mesoporous titanium dioxide nanoparticles (MTNs) can also absorb ultraviolet light to produce ROS and further kill the MDR tumor (Guo et al., 2018) (Table 5).

**Table 5.** Examples of studies overcoming MDR reversing via Photo therapy.

Type	Chemotherapeutic drug	Mechanism	Cell line	Refs
AuNP@mSiO_2_-DOX-FA	DOX	PTT	MCF-7/ADR	(Wu et al., 2019)
Cu_2-x_Se-based photothermal vector	TP-DOX	PTT	MCF-7/ADR	(Ji et al., 2021)
DOX-PCM@MCN-SLPD	DOX	PTT	MCF-7/ADR	(Hussain & Guo, 2019)
_ADL_Au@CuS YSNPs	DOX	PDT (Au NRs), NO	MCF-7/ADR	(Wang et al., 2019a)
Ru^II^-Pt^IV^ conjugate	Platinum (IV)	PDT (Ru(II) moieties)	A2780 ADR	(Karges et al., 2020)
Pt(IV)/Ru(II) bimetallic polymer	Cisplatin	PDT (Ru(II) moieties)	A549/DDP	(Zeng et al., 2020)
PpIX-Ahx-PEG_8-d_(KLAKLAK)_2_-GRGD/DOX	DOX	PDT (Protoporohyrin IX)	MCF-7/ADR	(Han et al., 2016)
DTX/Ce6-KNPs	DTX	PDT (Ce6)	HeLa-R	(Gaio et al., 2019)
UCNPs-loaded PEG-Pt(IV) nanoparticles	Platinum (IV)	PDT (Ce6)	HeLa (hypoxia)	(Xu et al., 2018a)
iNGR-VP-NA-DTX	DTX	PDT (Verteporfin)	HCT-15	(Jiang et al., 2019)
DEB/TQR@PMP micelles	PTX	PDT (DEB-BDTO)	SKOV-3/MDR	(Zhen et al., 2019)
P1-EPO NPs	2-chloro-4-nitroniline	PTT-PDT (DMN-EPO, BDP)	Hela (hypoxia)	(Li et al., 2021b)
Cetuximab-IR700 conjugates	Cetuximab	IR700	SBC-3/CDDP	(Takahashi et al., 2021)
GO-PAMAM-Poloxamer 188	DOX	PTT-PDT (ICG)	MCF-7/ADR	(Wang et al., 2019b)
γ-PGA-g-PLGA & cholesterol-PEG	DOX	PTT-PDT (ICG)	MCF-7/ADR	(Chen et al., 2019a)
DOX/ICG@biotin-PEG-AuNC-PCM	DOX	PTT-PDT (ICG)	MCF-7/ADR	(Yu et al., 2017)
S-nitroso donor conjugated polymers	DOX	PTT-NO-CT	MCF-7/ADR	(Ding et al., 2019)
ADH-1-HA-MTN/DOX	DOX	PDT (MTNs)	A549/EMT	(Guo et al., 2018)
FA-PEG modified polydopamine nanoparticles	DOX	PTT, NO	MCF7/ADR	(Wei et al., 2019)
Copper-palladium alloy tetrapod nanoparticle	Cu/Pd nanoparticles	Chemo-PTT	MCF7/MDR	(Zhang et al., 2018b)
RC@TFC	Rapamycin	PDT (Ce6)	MDA-MB-231 (hypoxia)	(Liu et al., 2019a)
PDOX-loaded Ce6-conjuaged PDPA micelles	Pluronic prodrug of DOX (PDOX)	PTT-PDT (Ce6)	MCF-7/ADR	(Wang et al., 2016c)

**Table 6.** Examples of studies overcoming MDR reversing via influence autophagy flow.

Type	Chemotherapeutic drug	Mechanism	Cell line	Refs
Inducing autophagy				
CS/PAA/VP-16@TPGS/PLGA NPs	Etoposide	Inducing apoptosis and autophagy	A549/DDP	(Wang et al., 2016d)
Zinc oxide nanoparticle (ZONs)	DOX	ZONs	MCF-7/ADR	(Hu et al., 2019)
Inhibiting autophagy				
Apolipoprotein A1-Modi Liposome	DOX	chloroquine (CQ) and LY294002	KBV	(Wang et al., 2014a)
PEG-PLA nanoparticles	DOX	chloroquine (CQ)	MDA-MB-231	(Sun et al., 2016)
PLGA/TPGS NPs	DOX	Chloroquine	A549/Taxol	(Sun et al., 2019)
PEO–PPO–PCL/TPGS micelles	DTX	chloroquine	MCF-7/ADR	(Shi et al., 2015)
Asymmetry-membrane liposomes	PTX	GAPDH-siRNA	HeLa and MCF-7 (hypoxia)	(Guan et al., 2017)
Nanoprodrug platform	Pt(IV)-peptide-	Beclin1 siRNA	A549/DDP	(Lin et al., 2019)
HP/Si-D Nanoparticles	DOX	ATG7 siRNA	A549/Dox	(Yang et al., 2020)
Au-PEG-SS-DOX NPs	DOX	AuNPs	HepG2-R	(Gu et al., 2012)

## 5. Autophagy and MDR

Autophagy is a lysosome-based degradative pathway, as well as a highly conserved process, initiated by the phagophore formation, which could degrade cytoplasmic materials (damaged organelles, obsolete proteins, and invading pathogens) and recycle energy to maintain homeostasis in cells (Li et al., 2017b). According to the degradative substances pathway, autophagy could be classified as macroautophagy, microautophagy, and chaperone-mediated autophagy. The term “autophagy” generally refers to macroautophagy because macroautophagy is primarily focused and widely researched (Sato et al., 2019). The normal authophagy flux process could be divided by three parts as follows phagophore assembly, autophagosome formation (and maturation), and autolysosome degradation. Firstly, autophagy is initiated by the cytoplasmic emergence of cup-shaped structures called phagophores and activated in limited growth conditions such as hypoxia, nutrient starvation, and chemoradiotherapy. Secondly, the phagophore continually elongates into a closed double-membrane structure and encapsulates cytoplasmic contents such as damaged organelles. Lastly, autolysosomes are formed by fusion with lysosomes to degrade the contents to maintain energetic homeostasis and viability. Accumulating evidence indicates that autophagy plays a vital role in the MDR development. Autophagy-related genes (Atgs) have been identified in several yeast and mammalian cells. Main Atgs include Atg12, Atg7, Atg 10, Atg 5, LC3, Atg4, and so on. The autophagy flux initiation symbol is the LC3B-I conversion into LC3B-II under Atg7 and Atg3. The LC3B-I is free of cytoplasm, but LC3B-II is directly bound to autophagosomes membranes. The SQSTM1/p62 is able to participate in various signaling pathways transduction, as well as autophagy flux, because of its special structure. The p62 level as an index, could reflect the autophagy process and be used to research the NDDS mechanism affecting autophagy.

### 5.1. Inducing autophagy

**Figure 6.**
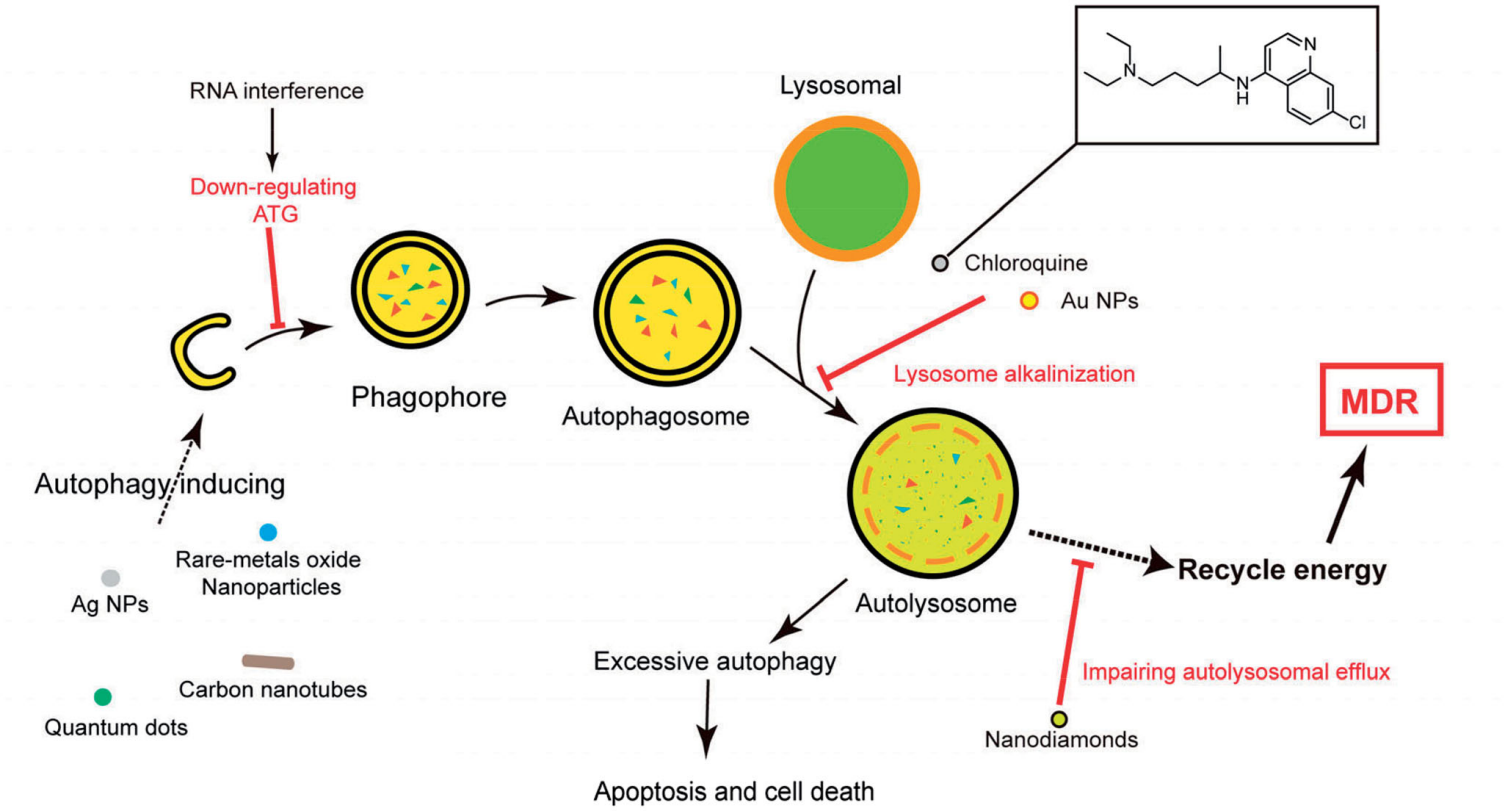
NP-mediated autophagy modulation mechanisms at different stages of autophaic flux.

Autophagy is an evolutionarily conserved catabolic process which degrades cytoplasmic organelles, proteins, and pathogens. However, excessive and sustained autophagy can promote apoptosis and kill MDR cancer cells by self-digestion (Li et al., 2017b). There are several materials that could be identified as autophagy inducers including quantum dots, Ag NPs, carbon nanotubes, cerium oxide, TiO2, and lanthanide oxide (Wan et al., 2018). The Atg5 plays an important role in autophagy flux. Zinc oxide nanoparticles were able to induce excessive autophagy due to promoting Atg5-regulated autophagy flux without the damage of autophagosome-lysosome fusion, which improve the chemotherapeutic agents’ efficacy for MDR cancer (Hu et al., 2019). The nanoparticles based on chitosan-poly(acrylic acid) delivered etoposide to combat MDR lung cancer, and then this nanosystems could enhance the antitumor effects by inducing apoptosis and autophagy (Wang et al., 2016d). Here, less NDDS utilized autophagy induction to resensitize chemotherapeutic drug but inhibiting autophagy received widespread concern (Figure 6).

### 5.2. Inhibiting autophagy

Autophagy could be seen as a prosurvival factor and has a role in the MDR development, which indicates that autophagy inhibitors might be one of the important strategies for overcoming MDR (Sun et al., 2011). Autophagy inhibition could reduce the breast CSCs “stemness” and elevate the sensitivity to chemotherapeutic agents (Sun et al., 2016). It is noteworthy that the MDR development caused by autophagy might be P-gp independent (Sun et al., 2015). The therapeutic strategies combining chemotherapeutic drug and autophagy inhibitors have been a common method in reversing MDR.

The Beclin1 gene (BECN1) is a mammalian-specific ATG6 homology, which could activate autophagic flux by forming the PI3K complex. Doxorubicin-hydrazone-caproyl-maleimide (DOX-EMCH) was simultaneously delivered with autophagy-inhibiting siBeclin1 to increase the tumor cell sensitivity to the chemotherapeutic drug (Hu et al., 2022). Considering that ATG7 plays a significant role in the autophagy regulation, autophagy downregulation through siRNA interfering in the ATG7 expression could reverse the A549/DOX MDR (Yang et al., 2020). The GAPDH downregulation by RNAi is able to suppress the autophagy and ATP levels. Simultaneously, PTX codelivery induces apoptosis and decreases hypoxia tumors drug resistance (Guan et al., 2017). Chloroquine (CQ), as an autophagy inhibitor, can block the autophagosomes fusion with lysosomes, which is able to accumulate in lysosomes and alkalize lysosomes (Shi et al., 2015; Sun et al., 2019). Wang et al. used DOX-loaded dapoA1-masked liposome to overcome MDR combated with CQ and LY294002 (a synthetic PI3K inhibitors) and detected that the autophagy elimination would enhance the chemotherapy efficiency (Wang et al., 2014a). Sun et al. applied the block copolymer PEG_5k_-b-PLA_8k_ to deliver CQ and chemotherapeutic agents for breast CSCs, which shows excellent tumor inhibition effects (Sun et al., 2016). Nanodiamonds (NDs) that show nanoparticle autophagy inhibition can enhance arsenic trioxide therapy for solid tumors; meanwhile, the autophagy inhibition works through impairing NUPR1-mediated autolysosomal efflux (Cui et al., 2018). The AuNP treatment causes autophagosome accumulation through autophagic flux blockade via lysosome alkalinization (Ma et al., 2011). Nanomaterials have been in extensive research about inducing authophagy, and the cell fates treated by nanomaterials are prodeath or prosurvival through blocking different autophagy processes (Zhang et al., 2019). However, nanomaterials such as AuNPs were usually applied as carriers with anticancer drug. Few drug delivery systems, codelivery of metal nanoparticles and chemotherapeutic drugs, are designed to research autophagy for reversing MDR.

## 6. Conclusion and future prospect

The NDDS has been widely applied in reversing MDR. We searched for many papers about MDR in this field. Higher chemotherapeutic agent doses are strategies for MDR, but it often bring more serious side effects and damages to normal tissues. Targeting drug delivery systems could respond to several conditions (such as pH, enzyme, light, and eta) to release drug to increase the drug concentration inside the tumor and enhance therapeutic efficacy. Researchers preliminarily understood the MDR mechanism, such as drug efflux, and found the drug efflux target. Thus, they took the advantage of nanocarriers to bypass P-gp-mediated drug efflux. The P-gp is a major obstacle for treating drug-resistant cancer, and its inhibition has been proven to be a successful strategy to overcome MDR. The P-gp activity could be inhibited in two ways as follows: (1) the P-gp was able to be directly suppressed by the P-gp inhibitor, or the expression level of it was disturbed by RNA interfering technology. (2) Considering that the P-gp needs energy supply from ATP, targeting mitochondrion or other strategies that disturb energy metabolism have been widely applied in overcoming MDR. The drug resistance does not only have one reason (P-gp) but is also a multifactorial and complex phenomenon. We concisely put drug efflux into a factor called pump factors and put other nonclear factors (such as gene, metabolize, signal pathway, and eta) into nonpump factors. For solving nonpump factors, photothermal therapy and photodynamic therapy have been used to enhance chemotherapeutic efficiency and obtain great achievement in reversing MDR. Autophagy is one of the main focuses of attention in the past decade. The dual autophagy characters are interesting and charming, but more and more papers have pointed out that autophagy inhibition could be conducive in reversing MDR (Li et al., 2017b; Sun et al., 2015). We believe that autophagy will retain the research highlights role in the future.

Although significant advances have been achieved in drug discovery, nano-drug delivery system has an irreplaceable role in cancer treatment. Co-delivery is a significant advantage for nanotechnology, which can achieve synergistic effects to overcome drug-resistance. In recent years, siRNA molecule is used to target disease-related genes, but conventional gene carriers are low uptake efficiency and high cytotoxicity. Efficient delivery, as well as high biosafety, is an indispensable feature for nano-drug delivery system. Leqvio^®^ (Inclisiran) was approved by FDA as adjunct therapy in 2021, which suggested that gene therapy would play more and more important role in disease treatment. However, there are two challenges in siRNA delivery: Firstly, naked siRNA will cause immunogenicity when is exposed in blood; Secondly, siRNA is hard to enter into cells due to strongly negative charge of siRNA. The above challenges can be tactfully solved by NDDS, which can improve gene-silence effect as well as chemotherapeutic efficiency. The co-delivery of chemotherapeutic drug and siRNA by multifunctional nanoparticles has the potential to overcoming MDR. Besides, antibody-drug conjugate (ADC) attracts widespread attention in cancer treatment due to its high targeting and good clinical efficacy. ADC is mainly composed of three parts: Targeting site, Linker, and Cytotoxin. The approval of ADC drugs, represented by Kadcyla^®^, indicates that it’s a feasible strategy in clinic that chemotherapeutic drugs are covalent attached to multifunctional polymeric backbone for MDR tumor. Most notably, the first ADC drug (Mylotarg^®^) had been withdrawn in 2010 because it’s instability in blood and causes severe toxicity. (Mylotarg^®^ was re-approved in 2017.) Thus, multifunctional nano-carriers must ensure the stability and controllability, although more functions may mean better effectiveness. Compared with chemotherapy, phototherapy has particular advantages such as higher targeting and lower toxicity. Photoimmunotherapy is a novel therapy through combining biology/medicine and photoabsorber. Akalux^®^, composed of cetuximab and IRDye700DX, was approved in 2020, which suggested that phototherapy can effectively improve targeting of drug delivery in clinic when be integrated within nano-carriers. Meanwhile, the NDDS could be also seen as a tool to study the MDR mechanism or other fields.

Despite the major clinical breakthroughs emerging in cancer therapy, drug resistance inevitably appears in cancer treatments. In the future, NDDS would solve the drug resistance question and further improve patient therapy efficiency and decrease side effects. Eventually, we will supply patients to safer, more biocompatible, more available, and less toxic preparations. For the achievement that can be ceaselessly acquired, let us believe that NDDS is likely to achieve significant breakthrough in the tumor treatment field.
